# Revised Draft Genome Sequences of Rhodomicrobium vannielii ATCC 17100 and Rhodomicrobium udaipurense JA643

**DOI:** 10.1128/MRA.00022-21

**Published:** 2021-04-01

**Authors:** Eric M. Conners, Emily J. Davenport, Arpita Bose

**Affiliations:** a Department of Biology, Washington University in St. Louis, St. Louis, Missouri, USA; University of Delaware

## Abstract

Recent attempts to sequence regions of the Rhodomicrobium vannielii ATCC 17100 genome revealed discrepancies with the previously published genome. We report the revised draft genome sequences of the type strains Rhodomicrobium vannielii ATCC 17100 and *Rhodomicrobium udaipurense* JA643. These revisions will facilitate genetic studies of phototrophic metabolism in these bacteria.

## ANNOUNCEMENT

The genus *Rhodomicrobium* is represented by three species, R. vannielii, R. udaipurense, and R. lacus (1–3). *Rhodomicrobium* strains are microaerobic to anaerobic, Gram-negative, budding, freshwater, purple nonsulfur bacteria capable of photoheterotrophic and photoautotrophic metabolism, including phototrophic iron oxidation by *R. vannielii* and *R. udaipurense* (1, 4–8). To date, six *Rhodomicrobium* genome sequences are publicly available, including those of *R. vannielii* ATCC 17100 (GenBank accession number NC_014664.1) and *R. udaipurense* JA643 (JFZJ00000000). Recent attempts to amplify and sequence regions of the phototrophic iron oxidation (*pio*) three-gene operon using ATCC 17100 genomic DNA (gDNA) revealed discrepancies between the *pioA* nucleotide sequence in the published genome and our sequencing data. The previously published ATCC 17100 genome was assembled using Newbler v. 2.3, which performs poorly relative to similar assemblers (9, 10) and contains a bug that reduces its effectiveness (https://cals.arizona.edu/swes/maier_lab/kartchner/documentation/index.php/home/docs/newbler). The use of this assembler might account for the discrepancies we observed. Here, we resequenced the ATCC 17100 and JA643 genomes, as the JA643 assembly used ATCC 17100 as a reference.

The *R. vannielii* type strain ATCC 17100 was purchased from DSMZ (Leibniz Institute, Braunschweig, Germany). The *R. udaipurense* type strain JA643 was acquired from the University of Hyderabad (Hyderabad, India). The strains were saved immediately as freezer stocks and regrown for genomic DNA isolation. Cell cultures, prepared in sterile anaerobic Balch tubes, were grown in bicarbonate-buffered anaerobic freshwater medium (6) supplemented with 10 mM sodium acetate and purged with H_2_-CO_2_ (80%/20%) to ∼70 kPa in the headspace. The cultures were incubated without shaking at 30°C, at a 30-cm distance from a 60-W incandescent light bulb. Genomic DNA was isolated from logarithmic-phase cultures using the DNeasy blood and tissue kit following the manufacturer’s recommendations (Qiagen, Dusseldorf, Germany). Paired-end 150-bp Illumina sequencing libraries were prepared as follows: 500 ng of gDNA was fragmented using a Covaris E220 sonicator. The DNA was blunt ended and had an A base added to the 3′ ends, and Illumina sequencing adapters were ligated to the ends. The ligated fragments were amplified for eight cycles using primers incorporating unique dual-index tags. The fragments were sequenced on a NovaSeq 6000 S4 instrument (Illumina, Inc.) to >200× coverage for both ATCC 17100 and JA643. The read quality was assessed with FastQC v. 0.11.9 (11), and the reads were trimmed with Trimmomatic v. 0.38 (12). These reads were assembled *de novo* with CLC Genomics Workbench v. 10.1.2 (Qiagen Bioinformatics) (13). The draft genome sequences were quality assessed with QUAST v. 5.0.2 (14) and submitted for annotation to the NCBI Prokaryotic Genome Annotation Pipeline (15). The resequenced genomes were compared to the previous genomes with OrthoANI (16) and BLASTn (17). Default parameters were used for all software.

The genome statistics for the draft genome sequences are found in Table 1. Average nucleotide identity (ANI) analyses confirmed discrepancies with the previously published genomes. The revised ATCC 17100 (AB38) genome has an ANI value of 94.73% compared with the previously published genome, with an average aligned length of 2,222,458 bp, or 55.37% reference coverage. The revised JA643 (AB60) genome has an ANI value of 99.94% compared to JA643, with an average aligned length of 2,634,102 bp, or 75.29% reference coverage. BLASTn alignments show that AB38 shares 88%, 80%, and 89% identities with each of the three *pio* operon genes, respectively, compared to the previously published ATCC 17100 genome. Importantly, AB38 *pioA* sequencing products from cultures originating from freezer stocks that we prepared upon receipt of each strain and prior to whole-genome sequencing (WGS) share 100% identity with the revised genome, compared to 88% with the reference. BLASTn alignments between AB60 and JA643 revealed 100% identity for each of the *pio* operon genes. These revised draft genome sequences will facilitate future efforts to investigate the genetics underlying these organisms’ metabolic strategies.

**TABLE 1:** Genome statistics

Strain	No. of reads	Assembly size (bp)	Coverage (×)	No. of contigs	*N*_50_ (bp)	G+C content (%)	Total no. of genes
AB38	4,992,886	3,849,085	185	177	81,079	62.2	3,644
AB60	15,446,669	3,652,920	500	94	113,688	62.5	3,387

### Data availability.

The whole-genome shotgun projects for AB38 and AB60 have been deposited in GenBank under the accession numbers JAEMUJ000000000 and JAEMUK000000000, respectively. The raw sequencing reads for AB38 and AB60 have been deposited in the NCBI Sequence Read Archive under the accession numbers SRX9703844 and SRX9703096, respectively. The versions described in this paper are JAEMUJ010000000 and JAEMUK010000000.

## References

[1] Duchow E, Douglas HC. 1949. *Rhodomicrobium vannielii*, a new photoheterotrophic bacterium. J Bacteriol 58:409–416. doi:10.1128/JB.58.4.409-416.1949. 16561801 PMC385647

[2] G S, Kumar D, Uppada J, Ch S, Ch VR. 2020. *Rhodomicrobium lacus* sp. nov., an alkalitolerent bacterium isolated from Umiam Lake, Shillong, India. Int J Syst Evol Microbiol 70:662–667. doi:10.1099/ijsem.0.003813. 31661050

[3] Tushar L, Sasikala C, Ramana CV. 2014. Draft genome sequence of *Rhodomicrobium udaipurense* JA643^T^ with special reference to hopanoid biosynthesis. DNA Res 21:639–647. doi:10.1093/dnares/dsu026. 25117430 PMC4263297

[4] Oren A, Xu X-W. 2014. The family Hyphomicrobiaceae, p 247–281. *In* Rosenberg E, DeLong EF, Lory S, Stackebrandt E, Thompson F (ed), The prokaryotes: Alphaproteobacteria and Betaproteobacteria. Springer, Berlin, Germany.

[5] Whittenbury R, Dow CS. 1977. Morphogenesis and differentiation in *Rhodomicrobium vannielii* and other budding and prosthecate bacteria. Bacteriol Rev 41:754–808. doi:10.1128/BR.41.3.754-808.1977. 334156 PMC414022

[6] Ehrenreich A, Widdel F. 1994. Anaerobic oxidation of ferrous iron by purple bacteria, a new type of phototrophic metabolism. Appl Environ Microbiol 60:4517–4526. doi:10.1128/AEM.60.12.4517-4526.1994. 7811087 PMC202013

[7] Gupta D, Sutherland MC, Rengasamy K, Meacham JM, Kranz RG, Bose A. 2019. Photoferrotrophs produce a PioAB electron conduit for extracellular electron uptake. mBio 10:e02668-19. doi:10.1128/mBio.02668-19. PMC6831781 31690680

[8] Trentini WC, Starr MP. 1967. Growth and ultrastructure of *Rhodomicrobium vannielii* as a function of light intensity. J Bacteriol 93:1699–1704. doi:10.1128/JB.93.5.1699-1704.1967. 6025454 PMC276670

[9] Kumar S, Blaxter ML. 2010. Comparing *de novo* assemblers for 454 transcriptome data. BMC Genomics 11:571. doi:10.1186/1471-2164-11-571. 20950480 PMC3091720

[10] Morrison SS, Williams T, Cain A, Froelich B, Taylor C, Baker-Austin C, Verner-Jeffreys D, Hartnell R, Oliver JD, Gibas CJ. 2012. Pyrosequencing-based comparative genome analysis of *Vibrio vulnificus* environmental isolates. PLoS One 7:e37553. doi:10.1371/journal.pone.0037553. 22662170 PMC3360785

[11] Andrews S. 2010. FastQC: a quality control tool for high throughput sequence data. http://www.bioinformatics.babraham.ac.uk/projects/fastqc/.

[12] Bolger AM, Lohse M, Usadel B. 2014. Trimmomatic: a flexible trimmer for Illumina sequence data. Bioinformatics 30:2114–2120. doi:10.1093/bioinformatics/btu170. 24695404 PMC4103590

[13] Qiagen. CLC Genomics Workbench 10.1.2. Qiagen, Redwood City, CA.

[14] Gurevich A, Saveliev V, Vyahhi N, Tesler G. 2013. QUAST: quality assessment tool for genome assemblies. Bioinformatics 29:1072–1075. doi:10.1093/bioinformatics/btt086. 23422339 PMC3624806

[15] Tatusova T, DiCuccio M, Badretdin A, Chetvernin V, Nawrocki EP, Zaslavsky L, Lomsadze A, Pruitt KD, Borodovsky M, Ostell J. 2016. NCBI Prokaryotic Genome Annotation Pipeline. Nucleic Acids Res 44:6614–6624. doi:10.1093/nar/gkw569. 27342282 PMC5001611

[16] Lee I, Kim YO, Park S-C, Chun J. 2016. OrthoANI: an improved algorithm and software for calculating average nucleotide identity. Int J Syst Evol Microbiol 66:1100–1103. doi:10.1099/ijsem.0.000760. 26585518

[17] Altschul SF, Gish W, Miller W, Myers EW, Lipman DJ. 1990. Basic local alignment search tool. J Mol Biol 215:403–410. doi:10.1016/S0022-2836(05)80360-2. 2231712

